# Feasibility of 3-Dimensional Visual Guides for Preparing Pediatric Zirconia Crowns: An In Vitro Study

**DOI:** 10.3390/ijerph17165732

**Published:** 2020-08-08

**Authors:** Ho Yeon Kang, Hyeonjong Lee, Yong Kwon Chae, Seoung-Jin Hong, Yun Yeong Jeong, Ko Eun Lee, Mi Sun Kim, Hyo-Seol Lee, Sung Chul Choi, Ok Hyung Nam

**Affiliations:** 1 Department of Pediatric Dentistry, School of Dentistry, Kyung Hee University, Seoul 02447, Korea; ghdus9658@naver.com (H.Y.K.); pedochae@gmail.com (Y.K.C.); pp5049@naver.com (Y.Y.J.); olivedlr@naver.com (K.E.L.); pedokms@khu.ac.kr (M.S.K.); snowlee@khu.ac.kr (H.-S.L.); pedochoi@khu.ac.kr (S.C.C.); 2 Department of Prosthodontics, Dental Research Institute, Dental and Life Science Institute, School of Dentistry, Pusan National University, Yangsan 46241, Korea; prostho.hjlee@gmail.com; 3 Department of Prosthodontics, School of Dentistry, Kyung Hee University, Seoul 02447, Korea; ssabock@hanmail.net

**Keywords:** computer-assisted three-dimensional imaging, crowns, deciduous tooth, dental materials, zirconium

## Abstract

This study evaluated the feasibility of a tooth preparation guide for prefabricated zirconia crowns (PZCs). Three-dimensional surface data for PZCs of the left maxillary primary first molar and left mandibular primary second molar were obtained using a model scanner. The tooth preparation data were digitally designed to harmonize with the adjacent teeth on the mixed dentition model and visualized using a color-coded map, which presents the required amount of tooth reduction. Twenty participants were recruited for preparing teeth with and without using the tooth preparation guide. The following three parameters were evaluated: tooth preparation time, harmony score, and amount of tooth reduction. The preparation time when using the guide was significantly reduced (*p* < 0.05), and a significant difference was observed in the harmony scores for the maxillary primary first molar preparation. Furthermore, the amount of tooth reduction was significantly different for both maxillary and mandibular primary molars (*p* < 0.05) in terms of the occlusal distal surface and buccal line angle in the maxillary primary first molars, and the smooth surfaces, proximal surfaces, and mesial line angles in the mandibular primary second molars. Thus, the results suggest that a tooth preparation guide could facilitate better tooth preparation for PZCs.

## 1. Introduction

Early childhood caries (ECC) refers to the presence of one or more decayed (lesions where a cavity has been formed or not), missing (due to caries), or filled tooth surfaces in the primary teeth of children aged 71 months or less [1]. It generally occurs and quickly spreads to multiple teeth, causing severe destruction to the tooth structure [2]. Therefore, such primary teeth require full-coverage restorations.

Traditionally, stainless steel crowns (SSCs) have been used for the full-coverage restoration of primary teeth; these are considerably different from the typical crown materials for permanent teeth. First, because SSCs are of pre-made sizes, the dentists must remove the carious enamel and dentine, select an appropriate crown size for the patient, and prepare the tooth to fit this crown size [3,4]. Second, as the SSCs are directly seated on the teeth without impression-taking and laboratory processes, the dentists need to prepare the teeth multiple times to ensure an adequate fit between the SSCs and the teeth [5,6]. Third, while the SSCs are seated to form retention based on their elasticity, and the crimping process can improve retention, they may exhibit lower marginal accuracy [7]. Finally, their silver color restricts the SSCs from meeting the esthetic needs of children and their guardians [8].

To address this concern, prefabricated zirconia crowns (PZCs) have been recently developed and marketed, which are more esthetic and biocompatible than the SSCs [9,10]. However, owing to the intrinsic physical properties and morphological characteristics of the PZCs, the tooth preparation methods would have to be different from those used for SSCs [11]; for instance, as the PZCs have low ductility and malleability, the crimping process cannot be implemented. Consequently, they should require a passive fit, for which repetitive tooth preparations are required. This could lead to excessive reduction of the tooth structure [12,13]. Furthermore, as the inner and outer shapes of the PZCs are different from those of the SSCs, special care must be taken during tooth preparation. According to a study that analyzed the inner and outer shapes of the PZCs and SSCs, the thickness of the SSCs is constant at approximately 0.2 mm regardless of the regions, while that of PZCs varies in the range of 0.3–0.8 mm, depending on the regions [13].

As appropriate preparation is important for the long-term prognosis of the PZCs [14], the objectives of this study were as follows: (1) to develop a tooth preparation guide for PZCs using three-dimensional (3D) visualized data and (2) to evaluate the feasibility of tooth preparation.

## 2. Materials and Methods

### 2.1. Study Design

This study proposal was reviewed and approved by the Ethics Committee of Kyung Hee University Dental Hospital, Kyung Hee University. The participants of this study were 10 residents at the Department of Pediatric Dentistry of Kyung Hee University and 10 dental university students of the School of Dentistry, Kyung Hee University. The students were fourth-grade students who had undergone clinical practice for at least a year. Using the mixed dentition dentiform model (Simple Root Tooth Model; Nissin, Kyoto, Japan), the left maxillary primary first molars and the left mandibular primary second molars were prepared for the PZCs and were three-dimensionally evaluated.

### 2.2. Digital Design of Preparation Guide

On a mixed dentition dentiform model, tooth preparation guides for the PZCs were prepared using models of the left maxillary primary first molars and the left mandibular primary second molars, which were reconstructed using 3D digital technology and analyzed.

- Model A: Dentiform model;
- Model B: Digitally scanned PZC set model;
- Model C: Ideally prepared tooth model for PZC;
- Model D: Actually prepared tooth model for PZC.

**Figure 1:**
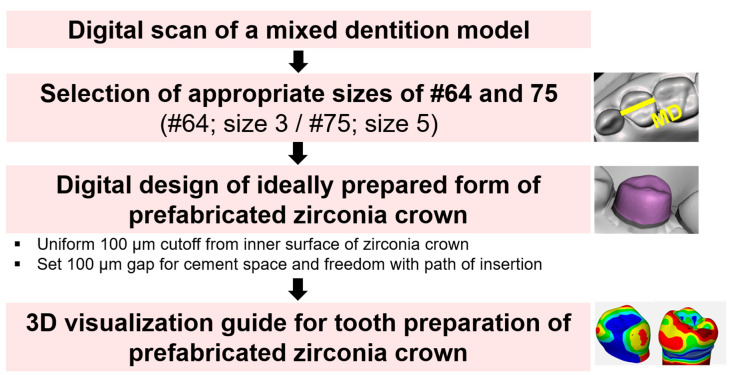
Digital workflow of the development of 3D-visualized data of the tooth preparation guide for prefabricated zirconia crowns (PZCs).

Scanned images of the mixed dentition dentiform model were obtained using a model scanner (Identica T500^®^; MEDIT Inc., Korea) (Model A). The sizes of the PZCs (NuSmile ZR; Orthodontic Technologies, Houston, TX, USA) that fit the mesiodistal sizes of the left maxillary primary first molar and the left mandibular primary second molar of Model A were selected using a 3D design program (Meshmixer; Autodesk, San Rafael, CA, USA) (Figure 1). The PZCs of sizes 3 and 5 were selected for the left maxillary primary first molars and left mandibular primary second molars, respectively.

**Figure 2:**
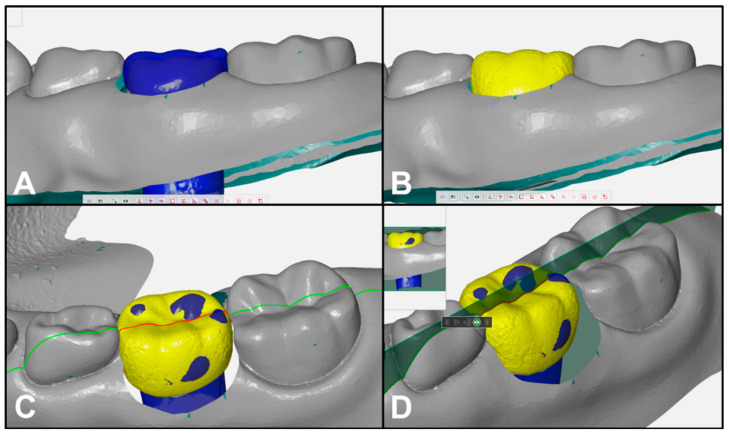
Digitally reconstructed models: (**A**) model A; (**B**–**D**) digital setup of PZC of the left mandibular primary second molar (Model B). The PZC was digitally aligned considering the marginal ridge level of the adjacent teeth and occlusion.

A low-viscosity silicone impression material (Imprint 3; 3M ESPE, St. Paul, MN, USA) was applied to the inner surface of the selected PZCs; the crown was fixed to a plaster base for easy scanning, and then the outer surface of the crown and the plaster base were scanned together using the model scanner. Next, the silicone impression material was carefully removed from the crown, and the inner surface of the crown and the plaster base were digitally scanned. Using a 3D design program (GOM Inspect 2018 software; GOM GmbH, Braunschweig, Germany), the 3D data of the PZCs for the left maxillary primary first molars and the left mandibular primary second molars were reconstructed and stored as separate files. The PZC files were three-dimensionally placed on Model A, considering the heights of the marginal ridges of the adjacent teeth and the adequacy of the occlusal relationship (Figure 2). After the crowns were positioned, the existing overlapping teeth were removed from the program.

**Figure 3:**
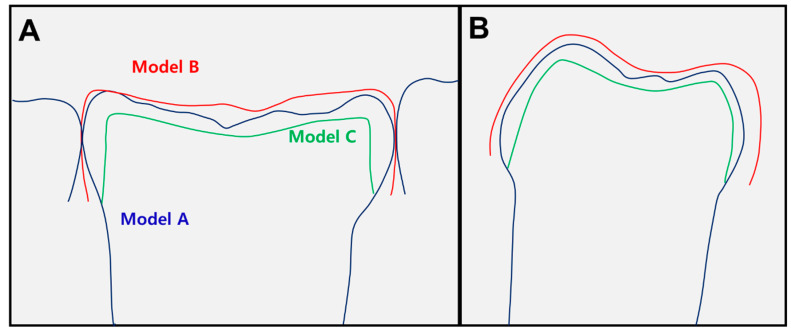
Superimposition of digitally reconstructed models: (**A**) mesiodistal section view of a left mandibular primary second molar; (**B**) buccolingual section view. The blue, red, and green lines represent models A, B, and C, respectively.

The ideal tooth shape (Model C) was attained by uniformly cutting from the inner surface of the PZC to provide the space for cement and freedom for the path of insertion. The thickness of the uniform cut was set as 100 μm, based on the clinical acceptance criteria for cement thickness of zirconia crowns for permanent teeth (Figure 3) [15,16].

**Figure 4:**
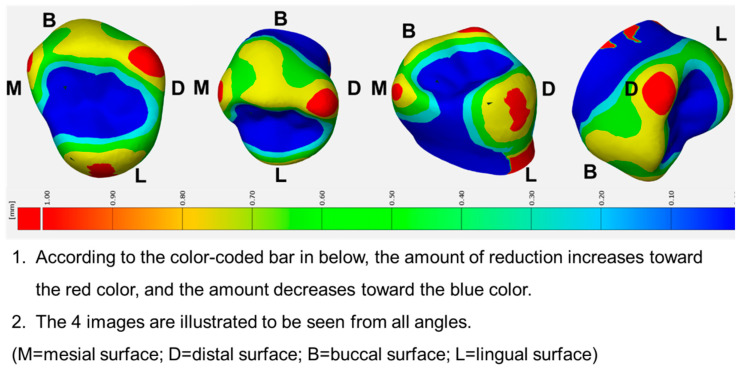
3D visualization of the tooth preparation guide for left maxillary primary first molar. The required amounts of tooth reduction are provided by a color-coded map. A detailed explanation of the figure was provided to the participants in Korean.

**Figure 5:**
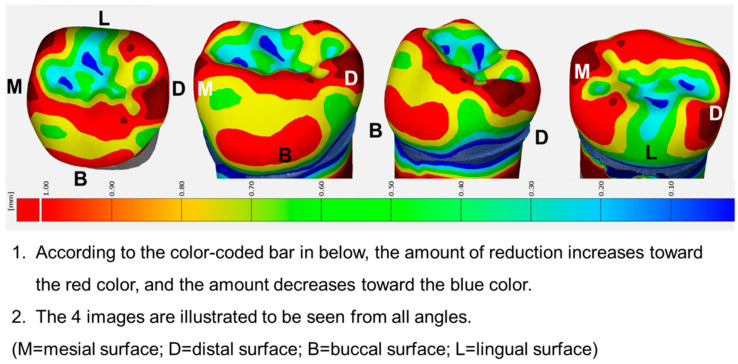
3D visualization of the tooth preparation guide of the left mandibular primary second molar. The required amounts of tooth reduction are provided by a color-coded map. A detailed explanation of the figure was provided to the participants in Korean.

Then, models A and C were superimposed, the differences between the three-dimensional (3D) distance analyzed, and a tooth preparation guide visualizing the detailed criteria for the amount of tooth reduction using color-coded maps produced (Figure 4 and Figure 5).

### 2.3. Evaluating the Feasibility of the Preparation Guide for PZCs

Each participant performed tooth preparation for PZCs on a dental phantom twice, i.e., with and without the guide. The tooth preparation time, harmony score, and differences in the amounts of tooth reduction were evaluated.

#### 2.3.1. Tooth Preparation Time

All participants prepared the left maxillary primary first molars and the left mandibular primary second molars for PZCs. The tooth preparation time was defined as the duration from placing a bur onto the typodont tooth until the corresponding PZC was completely seated on the tooth. Subsequently, 2 weeks later, the participants prepared the teeth for PZCs using the guide, and the tooth preparation time was measured again.

#### 2.3.2. Harmony Score with Adjacent Teeth

After completing the preparation, all participants delivered the PZCs. To evaluate the adequacy of the occlusal relationship, the differences in the marginal ridge heights between the adjacent teeth and the teeth fitted with the PZCs were investigated: a digital caliper (Mitutoyo, Tokyo, Japan) was used to determine the difference in the marginal ridge heights. Each difference was measured twice by an investigator and the measurements indicated favorable reproducibility (Intraclass correlation coefficient = 0.688 and 0.829 for the maxillary primary first molars and mandibular primary second molars, respectively).

The results were classified as follows:

- Point: Cases wherein the height differences between the marginal ridges of the PZC and the marginal ridges of the adjacent teeth were ≥ 2 mm on any of the mesial and distal side.
- Points: Cases wherein the height differences between the marginal ridges of the PZC and the marginal ridges of the adjacent teeth ranged between 1 mm and 2 mm on both the mesial and distal sides.
- Points: Cases wherein the height differences between the marginal ridges of the PZC and the marginal ridges of the adjacent teeth were < 1 mm on both the mesial and distal sides.

#### 2.3.3. Differences in the Amount of Tooth Reduction

**Figure 6:**
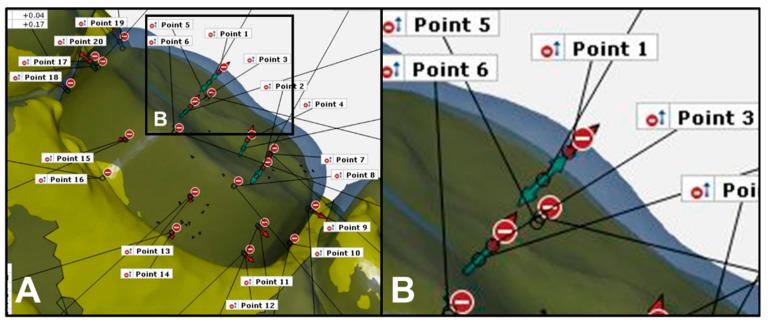
3D overlap of Model C (blue color) and Model D (yellow color): (**A**) reference points were set as follows: 2 points each in the occlusal cusp and occlusal fossa, 4 points on the proximal surfaces (2 points each on the mesial and distal surfaces), 4 points on the smooth surfaces (2 points each on the buccal-and lingual surfaces), and 8 points on the line angles (2 points per mesiobuccal, distobuccal, mesiolingual, and distolingual line angles); (**B**) high-magnification image of the square region of Point 1. The green arrow indicates the distance between the ideally and actually prepared teeth.

Two mixed dentition dentiform models (Model D) on which the teeth were prepared with and without the guide were scanned using the model scanner. Models C and D were superimposed on the computer image program. For Model C, a total of 20 region-wise reference points that were evenly and three-dimensionally distributed (four each on the occlusal, buccal, and lingual surfaces, and eight on the line angles) were marked, and the distances between the pairs of the corresponding points in Models C and D were measured (Figure 6). To compare the amount of tooth reduction, the results were evaluated using the root mean square (RMS) values. The mean and standard deviation of the RMS values obtained from the reference points corresponding to the occlusal surface, buccal surface, lingual surface, and line angles were calculated.

### 2.4. Statistical Analysis

The data were analyzed using a statistical program (SPSS for Windows, Version 20.0; IBM Corp., Armonk, NY, USA). As the results of the Shapiro–Wilk test demonstrated that the differences in the time taken for teeth preparation and the amount of reduction do not exhibit a normal distribution, the statistical analyses of these parameters were performed using the Wilcoxon signed-rank test. Furthermore, a post-hoc analysis of the differences in the amounts of reduction based on guide usage was performed using the Kruskal-Wallis test and Mann-Whitney test (post-hoc analysis). Cross-analyses were performed to statistically test the utility of the tooth preparation guide for the harmony with adjacent teeth during teeth formation, and Fisher’s exact test was conducted because more than 25% of the items had an expected frequency of 5 or lower. The *p*-values (< 0.05) indicated statistical significance.

## 3. Results

### 3.1. Tooth Preparation Time

Table 1 presents the time taken to prepare the teeth, with and without the tooth preparation guide: the preparation time for the maxillary primary first molars with and without the tooth preparation guide was 5.28 ± 1.75 min and 6.39 ± 2.40 min, respectively, which indicated that the time taken was reduced by the use of the tooth preparation guide (*p* < 0.0001). Similarly, the time taken to prepare the mandibular primary second molars with and without the tooth preparation guide was 4.98 ± 1.81 min and 5.63 ± 2.05 min, respectively, indicating shorter duration when the tooth preparation guide was used (*p* = 0.013).

### 3.2. Harmony Score

Table 2 shows that while the harmony scores for the maxillary primary first molars significantly varied with the use of the preparation guide (*p* = 0.044), those of the mandibular primary second molars did not exhibit any significant difference (*p* > 0.05).

### 3.3. Difference in the Amount of Tooth Reduction

**Figure 7:**
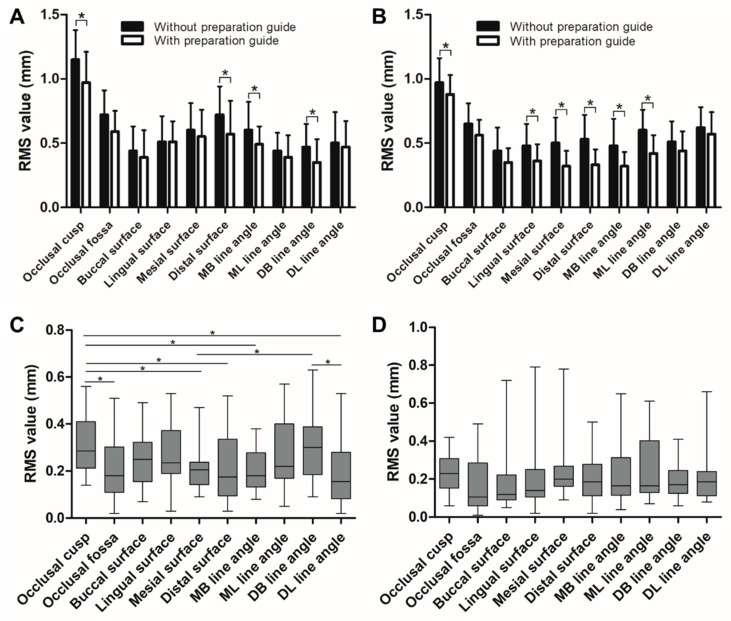
Comparisons of amount of tooth reduction: (**A**) root mean square (RMS) values for maxillary primary first molars, (**B**) RMS values for mandibular second molars, (**C**) differences in the amount of tooth reduction in maxillary primary first molars based on guide usage, and (**D**) differences in the amount of tooth reduction in mandibular second molars based on guide usage. Statistical significances are not observed among the surfaces and line angles. MB = Mesiobuccal, ML = Mesiolingual, DB = Distobuccal, DL = Distolingual; *statistically significant (*p* < 0.05).

Figure 7 shows that in the maxillary primary first molars, the amount of reduction achieved using the guide was smaller in the occlusal cusps, occlusal fossa, distal surface, mesiobuccal line angle, and distobuccal line angle regions (*p* < 0.05). Furthermore, significant differences were observed with guide usage among the surfaces and line angles (*p* < 0.05), especially in occlusal cusp and distobuccal line angle. However, in the mandibular primary second molars, the reduction amounts achieved by using the guide were smaller in the buccal surface, lingual surface, mesial surface, distal surface, mesiobuccal line angle, and mesiolingual line angle regions (*p* < 0.05); thus, there were no significant differences with guide usage among the surfaces and line angles.

## 4. Discussion

This study demonstrated that the time taken for teeth preparation was reduced by using the 3D visual guide for preparing both maxillary and mandibular molars. This could be attributed to the greater confidence of the participants using the guide. This finding was consistent with those of previous studies demonstrating that the use of a guide decreased the duration of dental surgery [17,18]. This time reduction for teeth preparation is clinically significant. First, it can reduce the total dental treatment time, which could improve the cooperation of pediatric patients, as the degree of cooperation is an important factor for increasing the success rate of treatment [19,20,21]. Second, the reduced tooth preparation time may reduce unnecessary exposure of the patients to drugs, especially for patients with difficulties in behavioral control (e.g., pediatric patients), conscious sedation, or general anesthesia [22,23].

The results of this study demonstrate that the participants could prepare teeth similar to Model C by using the tooth preparation guide. In clinical cases where molar teeth were restored using PZCs, the restoration was implemented after pulp therapy [9,24]. Clinically, the application of the guide can prevent excessive tooth reduction, thereby reducing unnecessary pulp exposure. Furthermore, the amount of reduction of the maxillary primary first molars was similar to that of Model C in the occlusal cusps, occlusal fossa, distal surface, mesiobuccal line angle, and distobuccal line angle regions, while that of the mandibular primary second molars was similar to that of Model C in the buccal surface, lingual surface, mesial surface, distal surface, mesiobuccal line angle, and mesiolingual line angle regions. Thus, the guide was not significantly helpful in the case of the lingual surface and lingual line angle region in the maxillary molars. However, for the mandibular molars, the guide was effective for most surfaces and regions (see Figure 7D). This phenomenon can be associated with securing the field of view; the participants experienced fewer difficulties while preparing the mandibular molars under direct view than while preparing the maxillary molars under indirect view via a dental mirror. These findings are in agreement with previous findings, which emphasize the importance of field of view on dental performance [25,26,27]. Moreover, the surface morphology of the PZCs affects this phenomenon. A study investigating the thicknesses of the PZCs reported that variations in thickness appear at line angles, which indicates that more appropriate tooth preparation is required in the line angle [13].

Thus, despite its several limitations, the results of this study provide useful insights in terms of dental education. Using digital technology, 3D-visualized preparation guides were created in advance, and tooth preparation was accurately evaluated. However, the number of participants could be considered as a limitation, in addition to the varying clinical experience and individual skill level among the participants. Moreover, a precise evaluation of the educational effects of this guide requires a cross-over study.

Furthermore, because this was an in vitro study, there were difficulties in accurately reproducing the sizes and shapes of the primary teeth of actual patients. Therefore, there may be differences from the tooth preparation guidelines for preparing the PZCs for actual patients. Therefore, to increase the clinical utility of the tooth preparation guide, diverse elements, such as variations in tooth morphology, tooth inclination, and convergence angle, should also be considered.

## 5. Conclusions

In conclusion, we developed 3D visual teeth preparation guides for PZCs and evaluated their feasibility. The use of the guide significantly reduced the tooth preparation time as well as the amount of unnecessary tooth reduction. Therefore, we believe that in the future, oral cavities of pediatric patients can be scanned to produce personalized guidelines for PZCs. Furthermore, the results of this study can be used as educational material for inexperienced clinicians, pediatric dentists, and dental students.

## Figures and Tables

**Table 1:** Time taken for tooth preparation.

Tooth	Without Preparation Guide (min)	With Preparation Guide (min)	*p*-Value
Maxillary first molar	6.39 ± 2.40	5.28 ± 1.75	<0.0001 *
Mandibular second molar	5.63 ± 2.05	4.98 ± 1.81	0.013 *

* Statistically significant (*p* < 0.05)

**Table 2:** Harmony scores.

Score	Without Preparation Guide *n* (%)	With Preparation Guide *n* (%)	*p*-Value
**Maxillary first molar**			
1	5 (25%)	0 (0%)	0.044 *
2	5 (25%)	5 (25%)	
3	10 (50%)	15 (75%)	
**Mandibular second molar**			
1	3 (15%)	1 (5%)	0.535
2	7 (35%)	6 (30%)	
3	10 (50%)	13 (65%)	

* Statistically significant (*p* < 0.05)
